# Gleanings

**Published:** 1897-04

**Authors:** 


### GLEANINGS.

A malignant and fatal outbreak of diphtheria in a section of West Philadelphia was noted to be associated with a similar condition among the cats equally severe in character and with marked throat lesions. By order of the Board of Health the cats were destroyed so far as possible.

A local outbreak of cerebro-spinal meningitis occurred in Kent County, Maryland, in March. Oat straw was supposed to have conveyed the germs.

At the Veterinary Department of the University of Pennsylvania, under the direction of State Veterinarian Pearson, several tuberculous cows are being treated with tuberculin and kept under the best hygienic conditions as a means of arriving at a better knowledge as to the value of this agent as a curative measure, and whether it is possible to use it in cases of mild tuberculosis with practical results.

Veterinarian Horton, of Providence, Rhode Island, reports the successful use of Sanmetto in a severe case of azoturia. The horse was down for forty-eight hours, regained his feet inside of seventy-two hours, and at the expiration of five days was placed at work.

A noted sheep raiser of Ohio, in the *Breeders' Gazette*, recommends the castration of lambs at the age of ten days. In docking, hot iron pincers are used to avoid hemorrhages.

The use of turpentine in burns is strongly extolled by Dr. McInnis in the New York *Medical Record*. Well sprinkled on a pad of absorbent-cotton, and applied, it aids the healing process very measurably. Endeavor to keep it applied to the burned surface only.

The most useful syringe for the administration of medicine by the mouth is a half-ounce hard-rubber one with a ring attachment to the piston-rod. The tongue may be held with one hand and the medicine syringed far back in the mouth, the ring aiding one by inserting the thumb in it, thus giving more force and not slipping off, as with a button-end rod. The amount given results in no loss, which often occurs in a larger-size syringe, which has the additional objection of not being so easily handled. Mouth-washes, tonics, mild stimulants, neutral fever mixtures, etc., are readily given this way without loss or interference with the taking of food or the worriment of drenching, which requires two persons.

Indiana possesses a law for liening colts, where the stallion is registered and licensed in accordance with the provision of the law.

But one importation of Percheron stallions to the United States was made in 1896.

Trichiniasis is rampant among the soldiers of the 104th Regiment at Chemnitz. Fifty-six of them are in the hospital, and fourteen have died as the result of eating pork of German or Bohemian raising.

One joint of beef out of every three consumed in London and the immediate district is American refrigerated beef.

A blunder occurred recently in one of our Eastern stock-yards where a detained storm-bound car-load of sheep was condemned by a local inspector for scab, which, on more careful and mature investigation by a veterinarian summoned by the shipper of the sheep, revealed only a loss of hair in some nineteen of the load, due to hair-pulling and biting incidental to the detention without food and water. This raises a serious question as to whether civil-service examination is yet broad enough to cover the needs in this service of our government, for a properly equipped inspector would hardly have committed this error, even if ever so careless. A government inspectorship for too many years was regarded as a sinecure or bounty, and as it was formerly obtained by political influence chiefly, and was only good for a term, it was considered unnecessary by the large proportion of those holding these positions to keep the dust from their text-books or read the journals so that every aspect of their work was fresh in their minds and they were equipped for every feature of their work. This, as well as disobedience of federal orders, must now be recognized as sufficient cause for dismissal from duty, and should be a lesson to all in the service that the government expects faithful work, and scientific in character also.

Robert T. Kneebs, on his second trial in Germany for bringing on the track the mare "Bethel" under the name of Nellie Kneebs, has again been convicted. He was sentenced to nine months imprisonment, to pay a fine of one thousand marks, to confiscation of his mare, and loss of civil privileges for two years. Prof. Eggling, the veterinarian sent to this country, testified against Mr. Kneebs.

A Flemington, N. J., cow, dying suddenly, an autopsy revealed pericarditis. Thirty-five nails from one to five inches long, sixteen stones about the size of walnuts, a carriage-bolt four inches long, and a padlock were found in the stomach. One of the nails had pierced the walls of the stomach and entered the muscular walls of the heart.

The Paterson, N. J., Veterinary Hospital has recently undergone a number of changes, making it more complete and perfect in its facilities. New canine-wards with special modern appliances; a bathtub in the equine department with hot- and cold-water connections; a specially fitted-up, large, padded stall for colic and other violent cases; enlargement of the pharmacy, and the introduction of electric lights throughout the building, are among the changes introduced by Veterinarian Lowe.

Veterinarian Bowles, late of Richmond, Va., is now a salesman in one of Pittsburg's queensware stores. Dr. John H. O'Brien is now practising human medicine and surgery in the Smoky City. Dr. Charles Spicer is a retouching artist in a photograph gallery of the latter city. They represent the Classes of 1886, 1892, and 1889, respectively, of the Ontario Veterinary College. Such are the shadows over the paths of veterinarians.

The employment of veterinarians in the preparation of popular articles on well-known diseases and lameness of horses for holiday numbers of the leading sporting and breeding journals and the almanacs of certain well-known live-stock periodicals is a hopeful sign of the times.

The present low prices of horses in the United States should be a means of supplanting the dog, cow, and ox in many parts of Europe where heretofore the high cost of horses led to the impounding of these animals as burden-bearers.

Fears are entertained by Western live-stock raisers that the reciprocal arrangements just entered upon between Canada and the United States whereby an interchange of cattle through ports where inspections can be made will militate against the Western cattle-grower and favor the Canadian raiser to this extent.

Captain H. I. Smith, of Iowa, a large owner of stock, is reported to have contracted tuberculosis from diseased cattle. A year ago State Veterinarian Stalker destroyed a number of tuberculous animals on Captain Smith's farm.

Chicago's horse-meat establishment ships a carload of horse corned-beef every three weeks to Rotterdam, Holland.

The *Breeders' Gazette* quotes the noted thoroughbred owner, John J. McCafferty, on long tails, as follows: "In the first place, a long tail on a horse is useful to brush off flies in hot weather. A horse whose tail has been banged will fret and become very restless in fly-time, when attacked by a fly, as he has no means of getting rid of it. Even in the stall he scrapes and frets until he is worn out, and many times a horse will rub all the hair off his hip-bones in his efforts to rub off some insect which is annoying him. With a long tail a horse can whisk it around and dislodge a fly from almost any part of his body. This is my first reason. Again, a horse is not nearly as liable to be cut down in a race when he has a long tail as he has when banged, for this reason: When a horse is running his tail sticks out similar to a flag in a high wind. This being the case, a horse coming from the rear cuts either to the inside or outside as soon as he catches a glimpse of a horse's tail in front of him. Consequently the longer the horse's tail the less liable is a horse to jump on him. Nervous horses always train better with long tails. They are generally thin-skinned and have short hair, consequently are bothered with insects more than a thick-skinned horse, and a long tail is almost a necessity to keep them from fretting and scraping until they become so weak and ill-tempered that they are not fit to race. I have found quite a difference between a thin-skinned horse and a thick-skinned horse. The latter class I have found very lazy, and have to be pushed to work. They also have a very heavy coat and do not answer to the whip and spur as readily as a thin-skinned horse. My reason for running horses high in flesh is the fact that they run stronger and run longer. A horse with plenty of flesh on his bones will run race after race without feeling it, while a horse keyed up to the highest pitch will surely go back after a race and has to be indulged before he is fit to race again. I am a great believer in running horses high in flesh."

